# Molecular epidemiology of *Neisseria gonorrhoeae* strains circulating in Indonesia using multi-locus variable number tandem repeat analysis (MLVA) and *Neisseria gonorrhoeae* multi-antigen sequence typing (NG-MAST) techniques

**DOI:** 10.1186/s12879-017-2940-5

**Published:** 2018-01-05

**Authors:** I Putu Yuda Hananta, Alje Pieter van Dam, Maarten Franciscus Schim van der Loeff, Mirjam Dierdorp, Carolien Marleen Wind, Hardyanto Soebono, Henry John Christiaan de Vries, Sylvia Maria Bruisten

**Affiliations:** 1 Academic Medical Center University of Amsterdam, Amsterdam, the Netherlands; 2 Public Health Laboratory,Public Health Service (GGD) of Amsterdam, Nieuwe Achtergracht 100, 1018 WT Amsterdam, the Netherlands; 3 Onze Lieve Vrouwe Gasthuis (OLVG) Hospital, Amsterdam, the Netherlands; 4 Amsterdam Infection and Immunity Institute (AI&II), Academic Medical Center, University of Amsterdam, Amsterdam, the Netherlands; 5 Department of Dermatology and Venereology Faculty of Medicine Universitas Gadjah Mada, Yogyakarta, Indonesia

**Keywords:** *Neisseria gonorrhoeae*, Indonesia, The Netherlands, Molecular epidemiology, Molecular typing

## Abstract

**Background:**

Control of gonorrhea in resource-limited countries, such as Indonesia, is mostly unsuccessful. Examining *Neisseria gonorrhoeae* (Ng) transmission networks using strain typing might help prioritizing public health interventions.

**Methods:**

In 2014, urogenital Ng strains were isolated from clients of sexually transmitted infection clinics in three Indonesian cities. Strains were typed using Multiple-Locus Variable Number Tandem Repeat (VNTR) Analysis (MLVA) and Ng Multi-Antigen Sequence Typing (NG-MAST) at the Public Health Service, Amsterdam, the Netherlands, and compared to Dutch strains collected from 2012 to 2015. Minimum spanning trees (MSTs) were constructed using MLVA profiles incorporating demographics and NG-MAST genogroups. A cluster was defined as ≥5 strains differing in ≤1 VNTR locus. The concordance between MLVA and NG-MAST was examined with the adjusted Wallace coefficients (AW).

**Results:**

We collected a total of 78 Indonesian strains from Yogyakarta (*n* = 44), Jakarta (*n* = 25), and Denpasar (*n* = 9). Seven MLVA clusters and 16 non-clustered strains were identified. No cluster was specific for any geographic area, risk group or age group.

Combined with 119 contemporary Dutch strains, 8 MLVA clusters were identified, being four clusters of Indonesian strains, two of Dutch strains, and two of both Indonesian and Dutch strains. Indonesian strains (79.5%) were more often clustered compared to Dutch strains (24.3%).

The most prevalent NG-MAST genogroups among Indonesian strains was G1407 (51.3%) and among Dutch strains was G2992 (19.3%). In Indonesian strains, the AW [95% confidence interval] for MLVA to NG-MAST was 0.07 [0.00–0.27] and for NG-MAST to MLVA was 0.03 [0.00–0.12].

**Conclusion:**

Indonesian *Ng* strains are more often clustered than Dutch strains, but show no relation with geographical area, risk group, or age group, suggesting a more clonal Ng epidemic in Indonesia. Some similar Ng strains circulate in both Indonesia and the Netherlands.

**Electronic supplementary material:**

The online version of this article (10.1186/s12879-017-2940-5) contains supplementary material, which is available to authorized users.

## Background

The prevalence of gonorrhea in Indonesia is high, especially among sex workers, men who have sex with men [MSM] and transwomen [1, 2]. For a successful control of gonorrhea and other sexually transmitted infections (STIs), especially in resource-limited countries, such as Indonesia, identification of high risk groups that need priority is required [3]. However, the overall risk to contract gonorrhea in the population is largely determined by the prevalence within transmission networks and the network characteristics [3–5].

In Indonesian STI clinics, transmission networks are traditionally identified by contact tracing using self-reported sexual history [6]. However, this approach is limited by recall bias, socially desirable responses, and privacy issues [7].

The use of molecular typing techniques may improve the quality and scope of transmission network identification by providing information on the spread of bacterial strains in different populations [8]. Various methods have been introduced to classify *Neisseria gonorrhoeae* (Ng) strains based on genotype [8–10].

Multiple-Locus Variable Number Tandem Repeat Analysis (MLVA) is a fast and robust method for molecular typing of Ng strains which is based on the variation in the number of tandem repeated DNA sequences in five loci of the bacterial genome [8, 9]. Ng Multi-Antigen Sequence Typing (NG-MAST) is a method using the combination of allele sequence variations of two gonococcal genes, *porB* and *tbpB,* to determine the strain type [8, 10].

No Indonesian Ng epidemiological study using MLVA or NG-MAST has yet been performed. We studied the transmission network of Ng in Indonesia by analyzing the distribution of Ng strains identified by MLVA and NG-MAST among clients of STI clinics in three major cities in Indonesia. In addition, we examined how Indonesian strains relate to other Ng strains by comparison with strains from Amsterdam, the Netherlands.

## Methods

### Study settings, population, and sample collection

This study was part of a larger study on gonorrhea epidemiology in Indonesia, which was partly reported elsewhere [2].

In 2014, clients of eight STI service facilities in three major cities in Indonesia (Jakarta, Yogyakarta, and Denpasar) were invited to participate in the study. A written informed consent was obtained from all participants. All participants were 16 years old or older at the day of recruitment. The participants completed a self-administered questionnaire regarding demographics, behavioral and clinical information. Based on the questionnaire data, three risk groups were defined: heterosexual males, heterosexual females, and a combined group of men who have sex with men and transwomen (MSM).

The clinicians collected urethral samples from males and transwomen, and endocervical samples from females. Primary culture and simple identification were performed at the laboratory of the Faculty of Biology, Universitas Gadjah Mada in Yogyakarta, Indonesia. Isolates were transferred on dry ice to the reference laboratory of the Public Health Service of Amsterdam, the Netherlands, where re-culture and complete identification were performed, as previously described [2]. The pure cultured Ng isolates were stored at −80 °C in beads (Microbank®, BioTrading, Mijdrecht, the Netherlands).

MLVA and NG-MAST have been used to type Ng strains at the reference laboratory in the past decade [5, 9]. A database containing molecular typing data of the strains, as well as characteristics of the patients from whom the strains were collected, was built in BioNumerics® version 7.5 (Applied-Maths, Austin, Texas, USA). To understand how Indonesian strains relate to circulating Ng strains in another setting, we used Dutch strains collected at the STI clinic of Amsterdam, the Netherlands, from two molecular epidemiology studies on azithromycin resistance [11], and ceftriaxone resistance [12]., All Dutch strains were fully susceptible for tested antibiotics as they acted as control strains in the two studies. The Dutch strains were collected between January 2012 and December 2015.

### Molecular typing using MLVA and NG-MAST

DNA lysates were prepared by boiling one stored bead seeded with cultured Ng isolates in 100 μL of phosphate buffered saline at 95 °C for 15 min.

MLVA was performed as described [9]. In short, DNA lysates were used for two different multiplex PCRs in a Bio-Rad C1000 PCR System (Bio-Rad, Hercules, California, USA) to amplify five variable number of tandem repeat sequence (VNTR) loci, i.e. VNTR04–03, VNTR04–10, VNTR07–02, VNTR30–01 and VNTR16–01. The PCR products were diluted 1:20 in water and 2 μl of each diluted sample was mixed with 18 μl of a 1:450 in water diluted GeneScan LIZ 500 size standard (Applied Biosystems, Foster City, California, USA). After heat denaturation for 5 min at 95 °C, the fragments were separated on an ABI 3130 Genetic Analyzer (Applied Biosystems, Foster City, California, USA) using the fragment analysis module. Sizing and calculation of the number of repeats of each VNTR were performed with GeneMarker® version 1.8 (SoftGenetics, LLC, State College, Pennsylvania, USA). An MLVA profile was a string consisting of integers that corresponds to the number of repeats found in the five VNTR loci [9]. This string was used for cluster analysis.

NG-MAST was also performed, as described [10]. In short, DNA lysates were used for two different PCR targets to amplify parts of the *porB* and *tbpB* genes in the Bio-Rad C1000 PCR System. The size of the amplimers in each PCR product was estimated using a QIAxcel® Advanced System (Qiagen, Hilden, Germany). PCR products with sufficient amplimers were transferred to the sequencing facility of the Academic Medical Center, University of Amsterdam, the Netherlands. An online international database of NG-MAST (www.ng-mast.net) was used as reference to determine allele numbers of *porB* and *tbpB* gene fragments, and to assign sequence types (STs). In addition, we checked the susceptibility profile of Indonesian strains against cephalosporins (ceftriaxone and cefixime), as previously reported [2], and examined its distribution by NG-MAST types.

### Genogrouping, cluster analysis, and statistics

We built a BioNumerics® database integrating participants’ characteristics and molecular typing data of Indonesian and Dutch reference strains.

Minimum spanning trees (MSTs) were constructed using MLVA profiles [9]. Two MSTs were constructed, i.e. an MST using data of Indonesian strains only and another MST using combined data of both Indonesian and Dutch strains. A cluster was defined as a group of ≥5 strains that differed in maximally one VNTR locus [9]. Clusters were assigned capital letters.

Based on NG-MAST STs, a genogroup was defined among strains that had one identical allele (*porB* or *tbpB*), and had another allele differing in ≤4 base pairs (bp) (*tbpB*) or ≤5 bp (*porB*), as described [13]. Genogroups were named after the most frequent STs within that genogroup. A genogroup consisting of ≥5 strains was considered as major genogroup.

Various participants’ characteristics, i.e. geographical location (city of the recruiting clinic), risk group, and age group, and NG-MAST genogroups were indicated in the MSTs.

We also examined the concordance between MLVA and NG-MAST by calculating the adjusted Wallace coefficient (AW) and its 95% confidence interval (CI) [14], using an online tool (www.comparingpartitions.info). For each country of strains collection, the AWs were calculated if the number of strains that belonged to one of MLVA clusters and one of the major NG-MAST genogroups was large >(20 strains) [14]. The AW of MLVA to NG-MAST showed the probability of strains within an MLVA cluster to belong to the same major NG-MAST genogroup. The AW of NG-MAST to MLVA showed the probability of strains within the same NG-MAST genogroup to belong to the same MLVA cluster. The AWs range from 0.00 (no association between the two techniques) to 1.00 (a complete association).

The statistical analyses were performed in STATA version 13 (Stata Corp., College Station, Texas, USA).

## Results

### Characteristics and distribution of Indonesian ng strains

From the 992 participants recruited in Indonesia, a total of 78 strains were successfully typed at the reference laboratory. The strains were from Yogyakarta (*n* = 44, 56.4%), Jakarta (*n* = 25, 32.1%), and Denpasar (*n* = 9, 11.5%) (Table 1). Strains were from heterosexual males (*n* = 5, 6.4%), heterosexual females (*n* = 30, 38.5%), and MSM (*n* = 43, 55.1%). Most of the participants (*n* = 46, 59.0%) were sex workers. Strains were mostly from participants aged 25–34 years (*n* = 34, 43.6%).

**Table 1:** Characteristics of *Neisseria gonorrhoeae* Strains Collected from Yogyakarta, Jakarta, and Denpasar, Indonesia (2014) and from Amsterdam, the Netherlands (2012–2015)^a^

Variables	Indonesian strains n (%)	Dutch strains n (%)
Total number of strains	78 (100.0)	119 (100.0)
City of strains collection		
Yogyakarta	44 (56.4)	0 (0.0)
Jakarta	25 (32.1)	0 (0.0)
Denpasar	9 (11.5)	0 (0.0)
Amsterdam	0 (0.0)	119 (100.0)
Year of strains collection		
2012	0 (0.0)	21 (17.6)
2013	0 (0.0)	32 (26.9)
2014	78 (100.0)	45 (37.8)
2015	0 (0.0)	21 (17.6)
Risk group of participants		
Heterosexual males	5 (6.4)	6 (5.0)
Heterosexual females	30 (38.5)	10 (8.4)
MSM^b^	43 (55.1)	103 (86.6)
Age group of participants		
16–24 years	22 (28.2)	28 (23.5)
25–34 years	34 (43.6)	39 (32.8)
35–44 years	16 (20.5)	24 (20.2)
≥ 45 years	6 (7.7)	28 (23.5)
MLVA clusters^c^		
Cluster A	16 (20.5)	0 (0.0)
Cluster B	7 (9.0)	1 (0.8)
Cluster C/D	13 (16.7)	1 (0.8)
Cluster E	10 (12.8)	0 (0.0)
Cluster F	7 (9.0)	0 (0.0)
Cluster G	9 (11.5)	0 (0.0)
Cluster H	0 (0.0)	10 (8.4)
Cluster I	0 (0.0)	5 (4.2)
Non-clustered strains^d^	16 (20.5)	102 (85.7)
NG-MAST genogroups^e^		
G1407	40 (51.3)	5 (4.2)
G21	0 (0.0)	15 (12.6)
G2400	0 (0.0)	7 (5.9)
G2992	13 (16.7)	23 (19.3)
G359	5 (6.4)	0 (0.0)
G5624	2 (2.6)	9 (7.6)
G9276	10 (12.8)	4 (3.4)
Other genogroups^f^	8 (10.3)	49 (41.2)
Unknown (data were missing/not available)	0 (0.0)	7 (5.9)

^a^Numbers were rounded to one decimal position. Therefore, the total percentage may not add up to be exactly 100% (may become 100.1% or 99.9%)

^b^men who have sex with men (including transwomen who did not undergo genital reassignment surgery;

^c^MLVA = Multiple-Locus Variable Number Tandem Repeat (VNTR) Analysis, a cluster was defined as a group of five strains or more that had a difference in at most one VNTR locus identified in a minimum spanning tree using a combined data of MLVA profiles of Indonesian and Dutch strains, assigned capital letters;

^d^a non-clustered strain was defined as a strain which was not grouped in a cluster;

^e^a grouping based on *Neisseria gonorrhoeae* Multiantigen Sequence Typing (see Material and Methods section);

^f^genogroups consisting of <5 strains

**Fig. 1:**
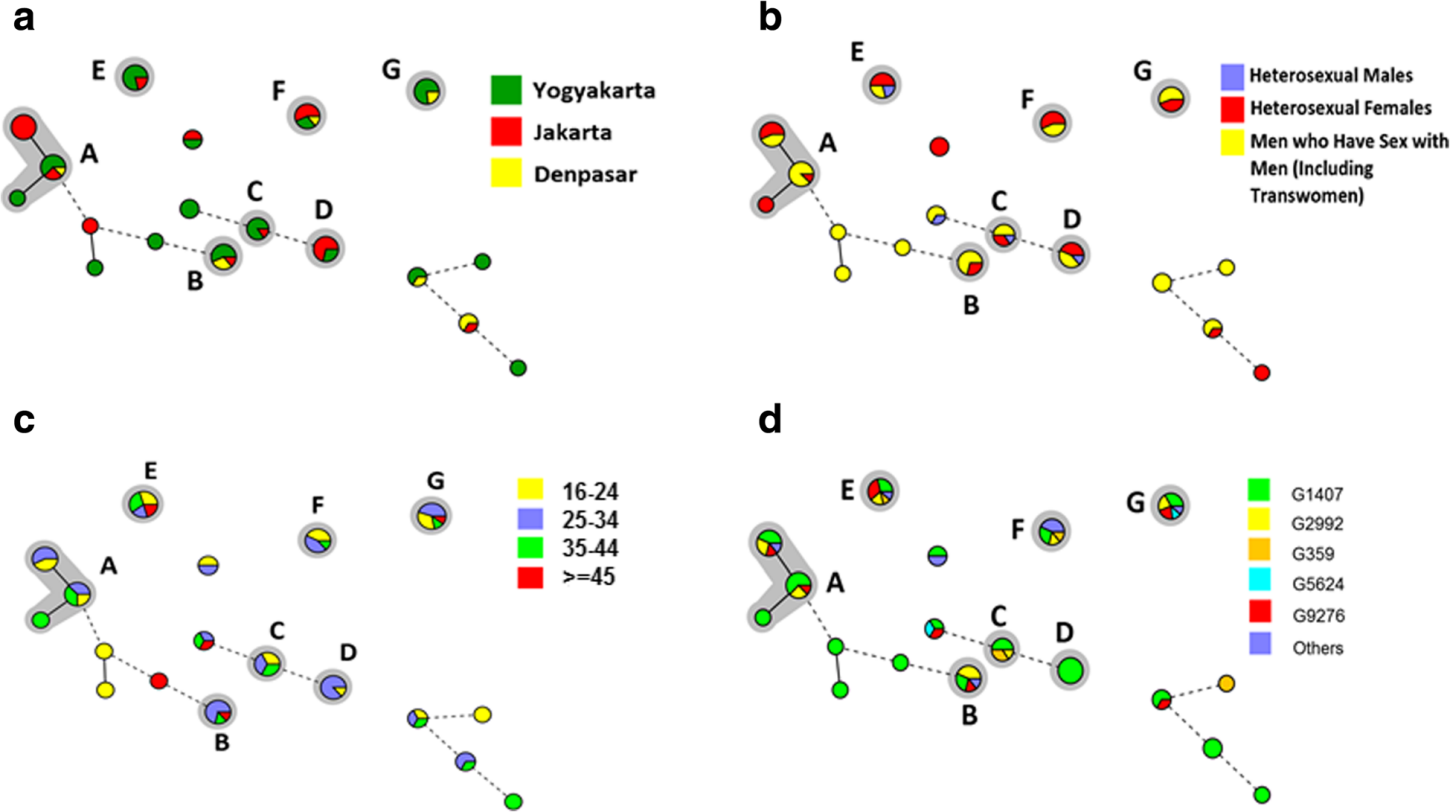
A minimum spanning tree (MST) based on Multiple-Locus Variable Number Tandem Repeat Analysis (MLVA) profile describing distribution of *Neisseria gonorrhoeae* (Ng) strains collected from Yogyakarta, Jakarta, and Denpasar, Indonesia in 2014. A circle represents strains that had identical MLVA profile; A gray halo surrounding circles represents a cluster, which was defined as a group of five or more strains that had a difference in at most one variable number tandem repeat (VNTR) locus, assigned capital letters (A-G); A solid line connecting circles represents a difference in one integer of MLVA profile; A dashed line connecting circles represents a difference in two integers of MLVA profile; *A grouping based on Ng Multi-antigen Sequence Typing (NG-MAST) (*see Material and Methods section*); **a** distribution of Indonesian Ng strains by city; **b** distribution of Indonesian Ng strains by risk group; **c** distribution of Indonesian Ng strains by age group; **d** distribution of Indonesian Ng strains by NG-MAST genogroup

Regarding the Indonesian strains, seven clusters (cluster A-G) and 16 non-clustered strains were identified (Fig. 1 and Additional file 1: Table S1). Cluster A (*n* = 16) was the largest cluster. Cluster C was relatively close to cluster D, differing in only two integers of their MLVA profiles. Other clusters differed in three or more integers from each other (Fig. 1).

Clusters B, C, E and G predominantly consisted of strains from Yogyakarta, whereas clusters A and F predominantly consisted of strains from Jakarta. In clusters A, B, and F, we observed strains from all three cities and no cluster was fully specific to one geographical area (Fig. 1a). Clusters A, B, and G predominantly consisted of strains from MSM and cluster F was predominantly from heterosexual females (Fig. 1b). In clusters C, D, and E, we observed strains from all risk groups, however (Fig. 1b). Clusters B and D predominantly consisted of strains from participants aged 25–34 years (which constituted the largest age group), but in clusters E and G, we observed strains from participants of all four age groups (Fig. 1c).

The 16 non-clustered strains were mostly isolated from Yogyakarta (*n* = 10, 62.5%) and from MSM (*n* = 11, 68.8%). The non-clustered strains were: three small groups each consisting of three strains with an identical MLVA profile, one group of two strains with an identical MLVA profile, and five singletons.

The most frequently occurring NG-MAST genogroup was G1407 (51.3%, Table 1) which was found in all Indonesian cities. We found no correlation between NG-MAST genogroups and geographical area, risk group, or age group (data not shown).

### Relation between Indonesian and Dutch ng strains

We included 119 Dutch contemporary strains as reference strains (Table 1). Most were from MSM (*n* = 103, 86.6%), and the remainder were from heterosexual males (*n* = 6, 5.0%) and heterosexual females (n = 10, 8.4%). Strains from participants who aged 25–34 years (*n* = 39, 32.8%) composed the largest group, as was the case for the Indonesian participants.

**Fig. 2:**
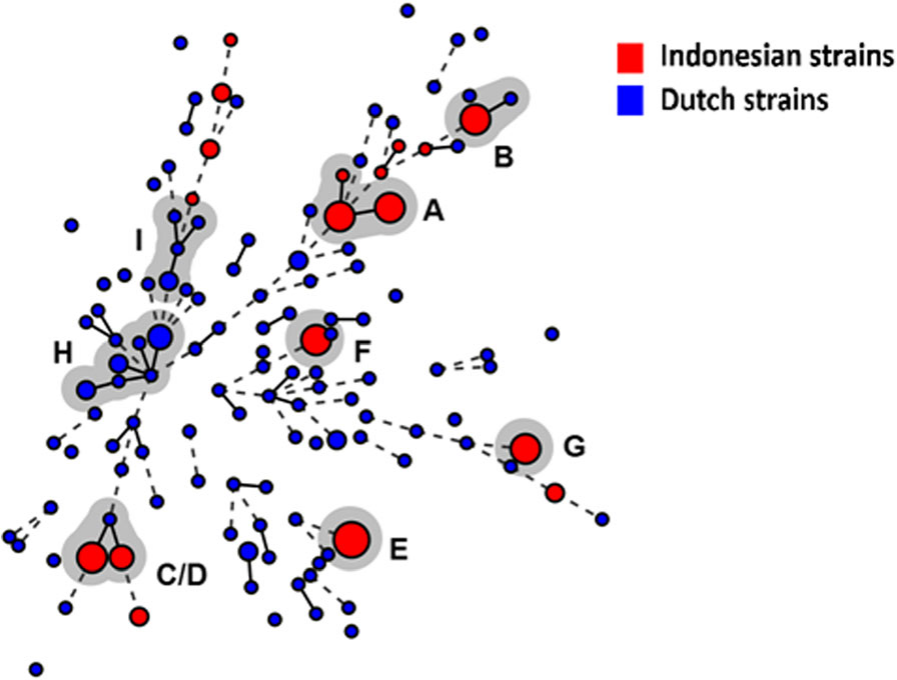
A minimum spanning tree (MST) based on Multiple-Locus Variable Number Tandem Repeat Analysis (MLVA) profile describing distribution of and relation between *Neisseria gonorrhoeae* (Ng) strains collected from Yogyakarta, Jakarta, and Denpasar, Indonesia (2014) and from Amsterdam, the Netherlands (2012–2015). A circle represents strains that had identical MLVA profile; a gray halo surrounding circles represents a cluster, which was defined as a group of five or more strains that had a difference in at most one variable number tandem repeat (VNTR) locus, assigned capital letters (**a**-**i**); a solid line connecting circles represents a difference in one integer of MLVA profile; a dashed line connecting circles represents a difference in two integers of MLVA profile

Regarding both Indonesian and Dutch strains, we identified eight clusters, including the seven clusters that had been identified in the MST using Indonesian data only (Fig. 2, Table 2). Cluster C/D was a consolidation of cluster C and D found in MST using Indonesian data only, and one Dutch singleton. Two clusters (B and C/D) consisted of both Indonesian and Dutch strains. Two clusters (H and I) consisted of Dutch strains only. We also identified 118 non-clustered strains (16 Indonesian and 102 Dutch strains).

**Table 2:** Characteristics and Distribution of *Neisseria gonorrhoeae* in a Minimum Spanning Tree Constructed Based on Multiple-Locus Variable Number Tandem Repeat Analysis (MLVA) Profile of Strains Collected from Yogyakarta, Jakarta, and Denpasar, Indonesia (2014) and from Amsterdam, the Netherlands (2012–2015)^a^

Variables	All strains	MLVA cluster^d^								Non-clustered strains^e^
		A	B	C/D	E	F	G	H	I	
	n (%)	n (%)	n (%)	n (%)	n (%)	n (%)	n (%)	n (%)	n (%)	n (%)
Number of strains^b^	197 (100.0)	16 (8.1)	8 (4.1)	14 (7.1)	10 (5.1)	7 (3.6)	9 (4.6)	10 (5.1)	5 (2.5)	118 (59.9)
City of strains collection^c^										
Indonesian cities	78 (39.6)	16 (100.0)	7 (87.5)	13 (92.9)	10 (100.0)	7 (100.0)	9 (100.0)	0 (0.0)	0 (0.0)	16 (13.6)
Amsterdam	119 (60.4)	0 (0.0)	1 (12.5)	1 (7.1)	0 (0.0)	0 (0.0)	0 (0.0)	10 (100.0)	5 (100.0)	102 (86.4)
Year of strains collection^c^										
2012	21 (10.7)	0 (0.0)	0 (0.0)	0 (0.0)	0 (0.0)	0 (0.0)	0 (0.0)	3 (30.0)	3 (60.0)	15 (12.7)
2013	32 (16.2)	0 (0.0)	1 (12.5)	0 (0.0)	0 (0.0)	0 (0.0)	0 (0.0)	5 (50.0)	1 (20.0)	25 (21.2)
2014	123 (62.4)	16 (100.0)	7 (87.5)	13 (92.9)	10 (100.0)	7 (100.0)	9 (100.0)	2 (20.0)	1 (20.0)	58 (49.2)
2015	21 (10.7)	0 (0.0)	0 (0.0)	1 (7.1)	0 (0.0)	0 (0.0)	0 (0.0)	0 (0.0)	(0.0)	20 (17.0)
Risk group or participants^c^										
Heterosexual males	11 (5.6)	0 (0.0)	0 (0.0)	2 (14.3)	2 (20.0)	0 (0.0)	0 (0.0)	1 (10.0)	0 (0.0)	6 (5.1)
Heterosexual females	40 (20.3)	6 (37.5)	2 (25.0)	5 (35.7)	5 (50.0)	4 (57.1)	4 (44.4)	0 (0.0)	0 (0.0)	14 (11.9)
MSM^#^	146 (74.1)	10 (62.5)	6 (75.0)	7 (50.0)	3 (30.0)	3 (42.9)	5 (55.6)	9 (90.0)	5 (100.0)	98 (83.1)
Age group of participants^c^										
16–24 years	50 (25.3)	5 (31.3)	0 (0.0)	4 (28.6)	3 (30.0)	3 (42.9)	3 (33.3)	1 (10.0)	1 (20.0)	30 (25.4)
25–34 years	73 (37.1)	7 (43.8)	6 (75.0)	8 (57.1)	2 (20.0)	3 (42.9)	4 (44.4)	5 (50.0)	3 (60.0)	35 (29.7)
35–44 years	40 (20.3)	4 (25.0)	1 (12.5)	2 (14.3)	3 (30.0)	1 (14.3)	1 (11.1)	2 (20.0)	1 (20.0)	25 (21.2)
≥45 years	34 (17.2)	0 (0.0)	1 (12.5)	0 (0.0)	2 (20.0)	0 (0.0)	1 (11.1)	2 (20.0)	0 (0.0)	28 (23.7)
NG-MAST genogroups^c, f^										
G1407	45 (22.8)	9 (56.3)	2 (25.0)	10 (71.4)	3 (30.0)	2 (28.6)	3 (33.3)	1 (10.0)	0 (0.0)	15 (12.7)
G21	15 (7.6)	0 (0.0)	0 (0.0)	0 (0.0)	0 (0.0)	0 (0.0)	0 (0.0)	0 (0.0)	0 (0.0)	15 (12.7)
G2400	7 (3.6)	0 (0.0)	0 (0.0)	0 (0.0)	0 (0.0)	0 (0.0)	0 (0.0)	0 (0.0)	0 (0.0)	7 (5.9)
G2992	36 (18.3)	4 (25.0)	3 (37.5)	1 (7.1)	2 (20.0)	1 (14.3)	2 (22.2)	8 (80.0)	4 (80.0)	11 (9.3)
G359	5 (2.5)	0 (0.0)	0 (0.0)	2 (14.3)	1 (10.0)	1 (14.3)	0 (0.0)	0 (0.0)	0 (0.0)	1 (0.9)
G5624	11 (5.6)	0 (0.0)	0 (0.0)	0 (0.0)	0 (0.0)	0 (0.0)	1 (11.1)	0 (0.0)	0 (0.0)	10 (8.5)
G9276	14 (7.1)	2 (12.5)	1 (12.5)	0 (0.0)	3 (30.0)	0 (0.0)	2 (22.2)	0 (0.0)	0 (0.0)	6 (5.1)
Other genogroups^g^	57 (28.9)	1 (6.3)	2 (25.0)	1 (7.1)	1 (10.0)	3 (42.9)	1 (11.1)	1 (10.0)	1 (20.0)	46 (39.0)
Unknown^h^	7 (3.6)	0 (0.0)	0 (0.0)	0 (0.0)	0 (0.0)	0 (0.0)	0 (0.0)	0 (0.0)	0 (0.0)	7 (5.9)

^a^Numbers were rounded to one decimal position. Therefore, the total percentage may not add up to be exactly 100% (may become 100.1% or 99.9%)

^b^numbers shown in bracket in this variable indicated the row percentage of number of strains in each cluster and non-clustered strains to the total number of combined Indonesian and Dutch strains;

^c^data for this variable were described in column percentage;

^d^a cluster was defined as a group of five strains or more which had a difference in at most one variable number tandem repeat (VNTR) locus, assigned capital letters (A-I);

^e^a non-clustered strain was defined as a strain which was not grouped in a cluster;

^f^a grouping based on *Neisseria gonorrhoeae* Multiantigen Sequence Typing (*see Material and Methods section*);

^g^genogroups consisting of <5 strains.

^h^NG-MAST data were not available in the database

Indonesian strains (*n* = 64, 79.5%) were more likely to be included in a cluster than Dutch strains (*n* = 17, 14.3%, *p* < 0.001). An identical MLVA profile of all strains in a cluster was observed in six out of seven Indonesian clusters, whereas the two Dutch clusters showed VNTR differences within the cluster, suggesting more diversity in the Dutch clusters.

The three most frequent NG-MAST genogroups among Indonesian strains were G1407 (*n* = 40, 51.3%), G2992 (*n* = 13, 16.7%), and G9276 (*n* = 10, 12.8%). Four Indonesian strains with *por* allele 908 and *tbpB* allele 2413 had an unidentified ST, yet these strains could be included in genogroup G1407. Indonesian strains that belong to ST1407 did not show decreased susceptibility against cephalosporins (ceftriaxone and cefixime), as we previously reported [2]. NG-MAST data were available for 112 out of 119 Dutch strains (NG-MAST was unsuccessful in 7 strains). The three most frequent genogroups among Dutch strains were G2992 (*n* = 23, 19.3%), G21 (*n* = 15, 12.6%), and G5624 (*n* = 9, 7.6%) (Table 1).

In 55 Indonesian strains that could be included in an MLVA cluster and a major NG-MAST genogroup, the AW [95% CI] for MLVA to NG-MAST was 0.07 [0.00–0.27] and for NG-MAST to MLVA was 0.03 [0.00–0.12]. The AWs in Dutch strains were not calculated because of a small sample size (n = 13 strains).

## Discussion

Here we describe Ng strains isolated from individuals living in three major Indonesian cities. A clustered pattern of Ng strains with no specific distribution regarding geographical location, risk group, or age group was observed. In addition, we found that most Indonesian strains were different from Dutch strains circulating in Amsterdam, the Netherlands, within overlapping time periods. Indonesian strains were more often clustered and therefore more clonal.

Several pathogen and individual factors may explain our findings. First, the finding of similar strain types circulating across different groups may be caused by the strain virulence and transmissibility [15, 16]. Strains with a higher level of virulence, e.g. those showing antimicrobial resistance or other high virulence characteristics, are more likely to survive and circulate more widely in the population [17–21]. Strains with resistance to third generation cephalosporins were so far not found in Indonesia [2], not even among ST1407 strains. This is in contrast to the situation in Europe, where fully susceptible ST1407 strains do occur, but the modal MIC of ST1407 strains to ceftriaxone is markedly increased [13].

Host and network factors also contribute to the pattern of strains circulating in the population [3, 4]. The finding of clusters that had no specific geographical location, risk group and age group distribution, may be associated with a disassortative sexual mixing which would enlarge the transmission network. Disassortative sexual mixing may be observed among individuals living in urban settings or among bridging populations (e.g. men who have sex with both men and women) [22–25].

A number of strains from patients in Indonesia were identified by active case finding in risk groups, whereas strains from Amsterdam were cultured from patients visiting the outpatient clinic. Active case finding may lead to identification of more epidemiologically related patients, and may in part explain the high frequency of clustering among Indonesian strains. However, this does not explain why clusters comprise strains from different cities.

Concordance between MLVA and NG-MAST was very low in Indonesian strains, much lower than was reported in previous studies [26, 27]. The number of studies comparing both MLVA and NG-MAST to type Ng strains is limited however, and the reported level of concordance between the two techniques varied greatly [26–28]. This could be caused by the different genetic regions of Ng targeted by these techniques [8]. Mutations or genetic translocation in these regions probably occur independently [15, 16]. In addition, discrepancy in the MLVA/NG-MAST concordance might relate to varying sample size and sample selection among studies [8, 26–28]. Also this MLVA has been validated using mainly European strains; it could be that by addressing different targets for Indonesian strains, this concordance could be improved.

MLVA is a relatively fast and cheap method compared to NG-MAST but needs a good local database. NG-MAST is a more robust technique with an internationally available database. To get maximum discriminatory typing power whole genome sequencing will be the best solution for typing but so far, pricing is still prohibitive certainly for developing countries [29].

We also observed a small overlap in MLVA clusters among Indonesian and Dutch strains. This could be the result of either an international spread of Ng strains or convergent evolutionary process that occurred in different geographical areas [15, 16].

Our study has some limitations. Indonesian and Dutch strains were collected through two distinct approaches. Data collection in Indonesia was performed only in 2014 (varying from 1 month to 5 months per participating clinic) and was focused on several specific risk groups, i.e. MSM, transwomen, and female sex workers living in an urban setting. Success of initial culture and of re-culture in the reference laboratory may be dependent on genetic background of the strains and on the timing of sample processing. For instance, strains from Yogyakarta, where the sample processing was quickly performed, were largely overrepresented (>56%). Therefore, our group of strains may not be totally representative for the Indonesian population under study.

We could not fairly compare the concordance of MLVA and NG-MAST between Indonesian and Dutch strains. The number of Dutch strains that could be included in one of the MLVA clusters and one of the major NG-MAST genogroups was too small for calculating the AWs. Small samples (*n* ≤ 20) results in wider 95% CI and lead to greater uncertainty in estimating the AWs [14].

The Dutch reference strains were all cultured immediately after sample collection from Ng positive visitors of only one STI clinic in Amsterdam, the Netherlands. The strains were collected regardless of risk group or age group criteria, yet the large majority of the strains (>87%) were nevertheless from MSM. These biases limit the comparison of Indonesian and Dutch strain data and limit the generalization of our findings to the population level.

Our study also has several strengths as it is the first to describe molecular epidemiology of Ng in Indonesia covering major cities and risk groups. It is also one of the few studies using both MLVA and NG-MAST techniques to describe Ng epidemiology. Finally, our study is able to show the relations of Ng strains circulating in two remotely separated settings (Indonesia and the Netherlands).

## Conclusion

We describe clusters of Ng strains collected from individuals living in an Indonesian urban setting and compared them with strains circulating in Amsterdam, the Netherlands. Indonesian Ng strains were more often clustered than Dutch strains, but showed no specific relation with geographical area, risk group, or age group. This finding suggests that a limited number of Ng clonal complexes circulated throughout Indonesia, at least in the three major cities in this study. This observation may challenge the intention to prioritize individuals of a certain geographical area, risk group, or age group in the sexual health program, e.g. the antimicrobial susceptibility screening. To describe the actual situation in the population, proportional samples from each group might be required in that program.

By comparing Indonesian and Dutch data, most of the strains appear to be unique for each country. However, some overlap in the MLVA clusters suggests that identical Ng strains might circulate in both Indonesia and the Netherlands. Whether this is an international spread or caused by a convergent evolutionary process, cannot be determined here.

Finally, the finding of a low concordance between MLVA and NG-MAST could be an important consideration regarding selection of typing techniques for future studies. With a better availability and affordable pricing, the application of whole-genome sequencing to investigate Ng transmission and antimicrobial resistance can be considered as an option [29].

## Additional file

Additional file 1: Table S1. Characteristics and Distribution of *Neisseria gonorrhoeae* in Minimum Spanning Tree Constructed Based on Multiple-Locus Variable Number Tandem Repeat Analysis (MLVA) Profile of Strains Collected from Indonesian Major Cities in 2014 (DOCX 25 kb)

## Abbreviations

AW: the adjusted Wallace coefficient
CI: confidence interval
DNA: deoxyribonucleic acid
MLVA: multi-locus variable number tandem repeat analysis
MSM: men who have sex with men
MST: minimum spanning tree
Ng: *Neisseria gonorrhoeae*
NG-MAST: *Neisseria gonorrhoeae* multi-antigen sequence typing
PCR: polymerase chain reaction
ST: sequence type
STI: sexually transmitted infections
VNTR: variable number tandem repeat
