# Supplementary material for: Molecular epidemiology of Neisseria gonorrhoeae strains circulating in Indonesia using multi-locus variable number tandem repeat analysis (MLVA) and Neisseria gonorrhoeae multi-antigen sequence typing (NG-MAST) techniques
Source: BMC Infect Dis. 2018 Jan 5;18:7. doi: 10.1186/s12879-017-2940-5 (PMC5755298; doi:10.1186/s12879-017-2940-5)
Supplement: Additional file 1: Table S1. — Characteristics and Distribution of Neisseria gonorrhoeae in Minimum Spanning Tree Constructed Based on Multiple-Locus Variable Number Tandem Repeat Analysis (MLVA) Profile of Strains Collected from Indonesian Major Cities in 2014 (DOCX 25 kb) [file 12879_2017_2940_MOESM1_ESM.docx]

Supplementary Table 1. Characteristics and Distribution of *Neisseria gonorrhoeae* in Minimum Spanning Tree Constructed Based on Multiple-Locus Variable Number Tandem Repeat Analysis (MLVA) Profile of Strains Collected

from Indonesian Major Cities in 2014^$^

| **Variables** | **All strains** | **Clustered strains*** | | | | | | | **Non-clustered strains**** |
| --- | --- | --- | --- | --- | --- | --- | --- | --- | --- |
|  |  | **A** | **B** | **C** | **D** | **E** | **F** | **G** |  |
|  | **n (%)** | **n (%)** | **n (%)** | **n (%)** | **n (%)** | **n (%)** | **n (%)** | **n (%)** | **n (%)** |
| **Number of strains**^†^ | 78 (100.0) | 16 (20.5) | 7 (9.0) | 6 (7.7) | 7 (9.0) | 10 (12.8) | 7 (9.0) | 9 (11.5) | 16 (20.5) |
| **City of strains collection**^‡^ |  |  |  |  |  |  |  |  |  |
| Yogyakarta | 44 (56.4) | 6 (37.5) | 4 (57.1) | 5 (83.3) | 2 (28.6) | 8 (80.0) | 2 (28.6) | 7 (77.8) | 10 (62.5) |
| Jakarta | 25 (32.1) | 9 (56.3) | 1 (14.3) | 1 (16.7) | 5 (71.4) | 2 (20.0) | 4 (57.1) | 0 (0.0) | 3 (18.8) |
| Denpasar | 9 (11.5) | 1 (6.3) | 2 (28.6) | 0 (0.0) | 0 (0.0) | 0 (0.0) | 1 (14.3) | 2 (22.2) | 3 (18.8) |
| **Risk group of participants**^‡^ |  |  |  |  |  |  |  |  |  |
| Heterosexual males | 5 (6.4) | 0 (0.0) | 0 (0.0) | 1 (16.7) | 1 (14.3) | 2 (20.0) | 0 (0.00 | 0 (0.0) | 1 (6.3) |
| Heterosexual females | 30 (38.5) | 6 (37.5) | 2 (28.6) | 2 (33.3) | 3 (42.9) | 5 (50.0) | 4 (57.1) | 4 (44.4) | 4 (25.0) |
| MSM^#^ | 43 (55.1) | 10 (62.5) | 5 (71.4) | 3 (50.0) | 3 (42.9) | 3 (30.0) | 3 (42.9) | 5 (55.6) | 11 (68.8) |
| **Age group of participants**^‡^ |  |  |  |  |  |  |  |  |  |
| 16-24 years | 22 (28.2) | 5 (31.3) | 0 (0.0) | 2 (33.3) | 1 (14.3) | 3 (30.0) | 3 (42.9) | 3 (33.3) | 5 (31.3) |
| 25-34 years | 34 (43.6) | 7 (43.8) | 5 (71.4) | 2 (33.3) | 6 (85.7) | 2 (20.0) | 3 (42.9) | 4 (44.4) | 5 (31.3) |
| 35-44 years | 16 (20.5) | 4 (25.0) | 1 (14.3) | 2 (33.3) | 0 (0.0) | 3 (30.0) | 1 (14.3) | 1 (11.1) | 4 (25.0) |
| ≥45 years | 6 (7.7) | 0 (0.0) | 1 (14.3) | 0 (0.0) | 0 (0.0) | 2 (20.0) | 0 (0.0) | 1 (11.1) | 2 (12.5) |
| **NG-MAST genogroups**^‡^**^** |  |  |  |  |  |  |  |  |  |
| G1407 | 40 (51.3) | 9 (56.3) | 2 (28.6) | 3 (50.0) | 7 (100.0) | 3 (30.0) | 2 (28.6) | 3 (33.3) | 11 (68.8) |
| G2992 | 13 (16.7) | 4 (25.0) | 3 (42.9) | 1 (16.7) | 0 (0.0) | 2 (20.0) | 1 (14.3) | 2 (22.2) | 0 (0.0) |
| G359 | 5 (6.4) | 0 (0.0) | 0 (0.0) | 2 (33.3) | 0 (0.0) | 1 (10.0) | 1 (14.3) | 0 (0.0) | 1 (6.3) |
| G5624 | 2 (2.6) | 0 (0.0) | 0 (0.0) | 0 (0.0) | 0 (0.0) | 0 (0.0) | 0 (0.0) | 1 (11.1) | 1 (6.3) |
| G9276 | 10 (12.8) | 2 (12.5) | 1 (14.3) | 0 (0.0) | 0 (0.0) | 0 (0.0) | 0 (0.0) | 2 (22.2) | 2 (12.5) |
| Other genogroups^^ | 8 (10.3) | 1 (6.3) | 1 (14.3) | 0 (0.0) | 0 (0.0) | 1 (10.0) | 3 (42.9) | 1 (11.1) | 1 (6.3) |

^†^numbers shown in bracket in this variable indicated the row percentage of number of strains in each cluster and non-clustered strains to the total number of Indonesian strains; ^‡^data for this variable were described in column percentage; *a cluster was defined as a group of five strains or more that had a difference in at most one variable number tandem repeat (VNTR) locus, identified in a minimum spanning tree using MLVA profiles of Indonesian strains, assigned capital letters (A-G); **a non-clustered strain was defined as a strain which was not grouped in a cluster; ^#^men who have sex with men (including transwomen who did not undergo genital reconstruction surgery); ^a grouping based on *Neisseria gonorrhoeae* Multiantigen Sequence Typing (*see Material and Methods section*); ^^genogroups consisting of <5 strains. ^$^*Numbers were rounded to one decimal position. Therefore, the total percentage may not add up to be exactly 100% (may become 100.1% or 99.9%).*
